# Translation and cross-cultural adaptation of the Telemedicine Satisfaction Questionnaire for use in Brazil

**DOI:** 10.36416/1806-3756/e20240030

**Published:** 2024-09-16

**Authors:** Maria E Leão, Soraya S Nohara, Ana C Fleury, José R Jardim

**Affiliations:** 1. Unidade de Reabilitação Pulmonar, Escola Paulista de Medicina/Universidade Federal de São Paulo, São Paulo (SP) Brasil.; 2. Disciplina de Pneumologia, Escola Paulista de Medicina/Universidade Federal de São Paulo, São Paulo (SP) Brasil.

**Keywords:** Telemedicine, Patient satisfaction, Surveys and questionnaires, Cross-cultural comparison, Smoking cessation

## Abstract

**Objective:**

To translate, cross-culturally adapt to Brazilian Portuguese, and evaluate the reliability of the Telemedicine Satisfaction Questionnaire (TSQ).

**Methods:**

This cross-sectional study involved patients from the Smoking Prevention and Cessation Center (PrevFumo) who participated in at least four of the eight scheduled remote meetings with the PrevFumo psychologist in 2020, 2021, or 2022. Participants were contacted by telephone and asked to answer the 14 questions of the TSQ three times at intervals of 7 or 10 days.

**Results:**

We assessed 53 patients (73.3% women). The mean age was 49.7 ± 10.2 years. The mean smoking history was 35.32 ± 24.8 pack-years. Of the 53 patients evaluated, 30.2% had completed high school or had some higher education, and 32.1% were classified as socioeconomic class B2 (A being the highest and E being the lowest). Forty-nine (92.5%) of the patients attended all eight meetings. The TSQ with only three answer options showed high reliability, with approximately 90% agreement after three applications. Patients were satisfied with telemedicine.

**Conclusions:**

The TSQ is rapidly applied, is easy to complete, and showed high reliability in our patient sample. Patients declared that they were satisfied with their telemedicine experience.

## INTRODUCTION

The Pan American Health Organization defines telemedicine as the remote, real-time delivery of health care services by any health professional using information and communication technologies to diagnose, treat, and prevent diseases.^1^

In Brazil, the use of telemedicine began in 1994, first being employed by cardiologists for remote electrocardiogram examinations.^2^ On December 27, 2022, law no. 14,510 amended law no. 8,080 (from September 19, 1990), to authorize and regulate the practice of telemedicine throughout the country. Telemedicine, known as *telessaúde* in Brazil, comprises the remote delivery of services related to all health professions regulated by competent agencies of the federal executive branch.

Several questionnaires assess patient satisfaction with telemedicine. The following are the most widely used: the Telehealth Usability Questionnaire, which assesses the feasibility of implementing telemedicine services^3^; the Telemedicine Satisfaction Questionnaire (TSQ)^4^; the Florida Patient Acceptance Survey^5^; the Telemedicine Satisfaction and Usefulness Questionnaire^6^; the Patient Satisfaction Questionnaire Short-Form^7^; the Patient Satisfaction with Physician^8^; the Service User Technology Acceptability Questionnaire,^9^ which assesses the beliefs of uses regarding the acceptability of telemedicine; and the Telehealth Satisfaction Scale, developed for patients with memory disorders.^10^

To our knowledge, there have been few studies evaluating patient satisfaction with telemedicine in Brazil, and none have used a translated, cross-culturally adapted questionnaire according to guidelines.^11^ ^,^ ^12^ Although Dias et al.^13^ used a questionnaire to evaluate the satisfaction of patients receiving telemedicine for the treatment of headaches, the description encompassed only the domains and possibilities of answers (“yes” and “no”), with no detailing of the questions. Severini et al.^14^ assessed the satisfaction of adult patients who used telemedicine, although their study lacked descriptions regarding how the questionnaire was adapted. Brandão et al.^15^ reported a satisfaction questionnaire to assess the acceptance and impact of telemedicine at a referral center for special immunobiologicals; however, the questionnaire questions were not described. Macharet et al.^16^ assessed the feasibility of telemedicine in urogynecology using only seven questions from a questionnaire originally consisting of 14. Dias et al.^17^ investigated the efficiency of real-time telerehabilitation in patients with Parkinson’s disease and found good patient satisfaction with telemedicine. However, their study sample comprised only 20 participants.

At the Paulista School of Medicine Smoking Prevention and Cessation Center (aka, PrevFumo), group orientation began remotely immediately after COVID-19 was declared a pandemic. However, when the pandemic was declared over, we had to decide whether the group orientation should return to in-person or continue being conducted remotely. To date, the in-person consultations have returned only for the initial visit. In this context, because of a lack of information regarding the satisfaction of our patients with remote group orientation, we felt compelled to assess their level of satisfaction before deciding whether to recommence the in-person group meetings. A literature review about satisfaction with telemedicine resulted in the choice of the TSQ because of its easy application and objective questions to assess the level of satisfaction with telemedicine and the reasons for the satisfaction.^4^ Nevertheless, before it could be used in Brazil, the TSQ would have to be translated and adapted to Brazilian culture.

The primary objective of this study was to translate, cross-culturally adapt, and validate the TSQ for use in Brazil. Secondary objectives were to analyze the relationships that satisfaction with telemedicine has with nicotine addiction, smoking history, education level, socioeconomic class, symptoms of anxiety, depressive symptoms, and quality of life.

## METHODS

Patients were invited by telephone to participate in this cross-sectional study. The study was approved by the Research Ethics Committee of the Federal University of São Paulo/Hospital São Paulo (Reference no: 64593722.0.0000.5505), in the city of São Paulo, Brazil. All participating patients gave written informed consent, either by e-mail or by text message. We included adult patients treated via the PrevFumo, regardless of sex and level of education, who participated in at least four of the eight scheduled remote group orientations.

Initially, the questionnaire was translated by one of the investigators who was fluent in English and Brazilian Portuguese.^11^ ^,^ ^12^ In the cross-cultural adaptation phase, the initial Brazilian Portuguese-language version was presented to 10 patients from our outpatient clinics, who discussed the words that best expressed what the questionnaire proposed. This version was further discussed by a multidisciplinary team, including a physiotherapist, a psychologist, a nurse, and a physician. The cross-culturally adapted version was then back-translated to English by another person and compared to the original English version to evaluate the similarity.^11^ ^,^ ^12^

After a given patient had been enrolled, the following data were collected from medical records of the initial consultation at PrevFumo: sociodemographic characteristics including age, gender, education, and socioeconomic class according to the Brazilian economic classification criteria^18^; smoking history; degree of nicotine addiction, determined with the Fagerström test^19^; respiratory symptoms; motivation to stop smoking; degree of anxiety, depression, or both, determined with the Hospital Anxiety and Depression Scale (HADS)^20^; and quality of life, determined with the World Health Organization Quality of Life Instrument, brief version (WHOQOL-BREF).^21^ The Brazilian economic classification criteria classify individual socioeconomic status into six classes from A (the highest) to E (the lowest). The Fagerström test comprises six questions with scores ranging from 0 (no addiction) to 10 (high addiction). The HADS has 14 questions that evaluate the probability of a diagnosis of anxiety or depression, with scores ranging from 0 to 21. The WHOQOL-BREF has 26 questions with a maximum total score of 130, higher values being associated with better quality of life.

The TSQ consists of 14 questions about satisfaction with telemedicine,^4^ answered on a five-point Likert scale (Table 1): 1 = strongly disagree; 2 = disagree; 3 = indifferent; 4 = agree; and 5 = strongly agree. To assess the reliability of the questionnaire, we recruited a sample of 50 participants to answer the 14 TSQ questions by phone call at three different time points, one-week apart.^22^ The same investigator conducted all three interviews, which ensured test-retest reliability.

**Table 1:** Telemedicine Satisfaction Questionnaire.

1.	I can easily talk to my healthcare professional. (Eu posso falar facilmente com o meu profissional da saúde)			
1- Strongly disagree	2- Disagree	3- Indifferent	4- Agree	5- Strongly agree
2.	I can hear my health care provider clearly. (Eu posso ouvir claramente o meu profissional da saúde)			
1- Strongly disagree	2- Disagree	3- Indifferent	4-Agree	5- Strongly agree
3	My health care provider is able to understand my health care condition. (Meu profissional da saúde pode entender minha condição de saúde)			
1- Strongly disagree	2- Disagree	3- Indifferent	4- Agree	5- Strongly agree
4.	I can see my health care provider as if we met in person. (Eu consigo ver meu profissional da saúde como se nós estivéssemos pessoalmente)			
1- Strongly disagree	2- Disagree	3- Indifferent	4- Agree	5- Strongly agree
5.	I do not need assistance while using the system. (Eu não preciso de ajuda quando estou usando o sistema da telemedicina)			
1- Strongly disagree	2- Disagree	3- Indifferent	4- Agree	5- Strongly agree
6.	I feel comfortable communicating with my health care provider. (Eu me sinto à vontade me comunicando com meu profissional da saúde)			
1- Strongly disagree	2- Disagree	3- Indifferent	4- Agree	5- Strongly agree
7.	I think health care provided via telemedicine is consistent. (Eu acho que a orientação sobre a saúde através da telemedicina é confiável)			
1- Strongly disagree	2- Disagree	3- Indifferent	4- Agree	5- Strongly agree
8.	I obtain better access to health-care services by using telemedicine. (Eu consigo fácil acesso ao serviço de saúde através da telemedicina)			
1- Strongly disagree	2- Disagree	3- Indifferent	4- Agree	5- Strongly agree
9.	Telemedicine saves me time traveling to hospital or a specialist clinic. (A telemedicina me economiza tempo em ir ao hospital ou a uma clínica especializada)			
1- Strongly disagree	2- Disagree	3- Indifferent	4- Agree	5- Strongly agree
10.	I receive adequate attention. (Eu consigo receber uma atenção adequada)			
1- Strongly disagree	2- Disagree	3- Indifferent	4- Agree	5- Strongly agree
11.	Telemedicine provides for my health care needs. (A telemedicina satisfaz a minha necessidade de cuidados da saúde)			
1- Strongly disagree	2- Disagree	3- Indifferent	4- Agree	5- Strongly agree
12.	I find telemedicine an acceptable way to receive health care services. (Eu acho a telemedicina um modo aceitável de receber serviços de cuidados à saúde)			
1- Strongly disagree	2- Disagree	3- Indifferent	4- Agree	5- Strongly agree
13.	I will use telemedicine services again. (Eu usaria usarei os serviços da telemedicina novamente)			
1- Strongly disagree	2- Disagree	3- Indifferent	4- Agree	5- Strongly agree
14.	Overall, I am satisfied with the quality of service being provided via telemedicine. (No geral, eu estou satisfeito com a qualidade do serviço sendo fornecida através da telemedicina)			
1- Strongly disagree	2- Disagree	3- Indifferent	4- Agree	5- Strongly agree

Statistical analyses were conducted using the R software, version 4.2.2.^23^ Data normality was verified with the Shapiro-Wilk test. Values are presented as absolute and relative frequencies (for categorical variables) or as mean and standard deviation (for continuous variables). Intraclass correlation coefficients (ICCs) were calculated in order to compare the reliability of TSQ responses among the three applications, and results were classified as poor (< 0.5), moderate (0.5-0.75), good (> 0.75-0.9), or excellent (> 0.9).^24^ For correlation analysis between the TSQ score and the nonparametric variables age, education, socioeconomic class, Fagerström test score, smoking history, anxiety, depression, attendance to the virtual education meetings, and quality of life, Spearman’s test was used. For comparison of the nonparametric variable sex, the Mann-Whitney test was used. There is no consensus on the interpretation of correlation values, but it is widely accepted that values under 0.4 should be considered indicative of a weak correlation. Values of p < 0.05 were considered statistically significant.

## RESULTS

The final sample consisted of 53 participants, 73.3% of whom were female (Table 2). The mean age was 49.7 ± 10.2 years. Of the 53 patients evaluated, 16 (30.2%) had completed high school or had some higher education. The most common socioeconomic class (in 32.1%) was B2 (29-37 points). A total of 92.5% of participants connected to all eight meetings. The mean total score of the WHOQOL-BREF was 12.3 ± 2.49 (51.9% ± 15.5%), and the mean total score of the Fagerström test was 5.81 ± 2.28 (39.6% were highly addicted). The mean smoking history was 35.3 ± 24.8 pack-years. On the HADS, the mean anxiety score was 10.8 ± 4.4 (45.3% had probable anxiety), whereas the mean depression score was 8.2 ± 3.7 (49.1% had probable depression).

**Table 2:** Sociodemographic and clinical characteristics of the participants.

Variable	(N = 53)
Age (years), mean ± SD	49.7 ± 10.0
Sex	
Female, n (%)	39 (73.6)
Male, n (%)	14 (26.4)
Weight (kg), mean ± SD	74.5 ± 20.3
Height (cm), mean ± SD	163.5 ± 9.9
Level of education, n (%)	
None or ≤ 9 years of schooling	7 (13.2)
High school graduate ± some college	22 (41.5)
College graduate ± graduate school	33 (45.3)
Socioeconomic class, n (%)	
A (45-100 points)	4 (7.6)
B (29-44 points)	25 (47.2)
C (17-28 points)	23 (43.4)
D-E (0-16 points)	1 (1.9)
Number of meetings attended, n (%)	
4	2 (3.8)
5	1 (1.9)
6	1 (1.9)
8	49 (92.5)
WHOQOL-BREF	
Total score, mean ± SD	12.3 ± 2.5
Percentage, mean ± SD	51.9 ± 15.5
Fagerström test	
Total score, mean ± SD	5.8 ± 2.3
Score of 0-4, n (%)	15 (28.3)
Score of 5-10, n (%)	38 (71.7)
Smoking history (pack-years), mean ± SD	35.3 ± 24.8
HADS anxiety score, mean ± SD	10.8 ± 4.4
0-7, unlikely, n (%)	14 (26.4)
8-21, likely, n (%)	39 (73.6)
HADS depression score, mean ± SD	8.17 ± 3.7
0-7, unlikely, n (%)	20 (37.7)
8-21, likely, n (%)	33 (62.3)

WHOQOL-BREF: World Health Organization Quality of Life Instrument, brief version; and HADS: Hospital Anxiety and Depression Scale.

### Reliability analysis

Table 3 presents the ICC between the three applications of the TSQ. Reliability was poor for 8 of the 14 questions. In addition, reliability values were better in the second and third interviews than in the first interview, probably because of the poor-to-moderate concordance found.

**Table 3:** Reliability analysis for the three applications of the Telemedicine Satisfaction Questionnaire.

Question	ICC	95% CI	p-value	Interpretation
1	0.488	0.325-0.640	0.550	Poor
2	0.469	0.306-0.623	0.639	Poor
3	0.599	0.440-0.732	0.103	Moderate
4	0.400	0.233-0.565	0.877	Poor
5	0.490	0.328-0.641	0.538	Poor
6	0.555	0.402-0.693	0.228	Moderate
7	0.416	0.251-0.579	0.835	Poor
8	0.582	0.433-0.714	0.132	Moderate
9	0.490	0.327-0.642	0.538	Poor
10	0.591	0.440-0.721	0.112	Moderate
11	0.591	0.435-0.723	0.118	Moderate
12	0.499	0.333-0.650	0.499	Poor
13	0.432	0.264-0.594	0.784	Poor
14	0.537	0.379-0.679	0.311	Moderate

ICC: intraclass correlation coefficient.

### Descriptive analysis for the grouped score with five answer options

Table 4 shows the frequency of combinations found in the three interviews, from which we considered a total of 742 answers (53 participants times 14 questions). The percentage of concordance for the answers “strongly agree” (number 5) and “agree” (number 4) in the three interviews was 46% and 16.7%, respectively. The following five most frequent combinations included only the answers “strongly agree” and “agree” (numbers 4 and 5).

**Table 4:** Descriptive analysis of grouped scores with five answer options in three interviews (N = 742) and the frequency of answer combinations.

Answers			Frequency of answer combinations
Interview 1	Interview 2	Interview 3	n (%)
5	5	5	343 (46.2)
4	4	4	124 (16.7)
4	5	5	92 (12.4)
4	4	5	35 (4.7)
5	4	4	25 (3.4)
5	4	5	21 (2.8)
4	5	4	17 (2.3)
2	4	4	13 (17.5)
5	5	4	10 (1.3)
4	3	4	7 (0.9)
3	4	4	7 (0.9)
4	4	2	4 (0.5)
3	4	5	4 (0.5)
2	5	5	4 (0.5)
2	4	3	3 (0.4)
3	3	4	3 (0.4)
2	5	4	3 (0.4)
2	4	5	3 (0.4)
3	5	5	3 (0.4)
2	3	3	2 (0.3)
3	3	3	2 (0.3)
4	3	3	2 (0.3)
4	4	3	2 (0.3)
2	2	4	2 (0.3)
5	2	5	2 (0.3)
2	2	2	1 (0.1)
2	4	2	1 (0.1)
4	5	2	1 (0.1)
4	2	3	1 (0.1)
5	3	3	1 (0.1)
3	4	3	1 (0.1)
1	5	3	1 (0.1)
4	5	3	1 (0.1)
2	3	4	1 (0.1)

Option 1: strongly disagree; Option 2: disagree; Option 3: indifferent; Option 4: agree; and Option 5: strongly agree.

### Descriptive analysis for the grouped score with three answer options

During the interviews, participants repeatedly reported not seeing differences between the answers “strongly disagree” and “disagree” and between “strongly agree” and “agree”. In this sense, we reanalyzed data considering “strongly disagree” and “disagree” as one answer and “strongly agree” and “agree” as another answer; thus, resulting in three options: “disagree” (number 1), “indifferent” (number 2), and “agree” (number 3). With this new classification, we observed that 89.9% of participants answered “agree” (number 3) in all three interviews (Table 5). 

**Table 5:** Descriptive analyses of grouped scores with three answer options in three interviews (N = 742) and the frequency of answer combinations.

Answers			Frequency of answer combinations
Interview 1	Interview 2	Interview 3	n (%)
3	3	3	667 (89.9)
1	3	3	23 (3.1)
2	3	3	14 (1.2)
3	2	3	7 (0.9)
3	3	1	5 (0.7)
1	3	2	4 (0.5)
3	2	2	3 (0.5)
3	3	2	3 (0.4)
2	2	3	3 (0.4)
1	2	2	2 (0.3)
2	2	2	2 (0.3)
1	1	3	2 (0.3)
3	1	3	2 (0.3)
1	1	1	1 (0.1)
1	3	1	1 (0.1)
3	1	2	1 (0.1)
2	3	2	1 (0.1)
1	2	3	1 (0.1)

Option 1: disagree; Option 2: indifferent; Option 3: agree.

### Correlations between TSQ score and sociodemographic variables

No association was found between the TSQ score and sex on the Mann-Whitney test (W = 320.5, p = 0.332). On Spearman’s correlation test, the TSQ score was not found to correlate with age (r = −0.18, p = 0.196), education (r = −0.17, p = 0.21), socioeconomic class (r = 0.21, p = 0.140), attendance to telemedicine educational meetings (r = 0.24, p = 0.080), nicotine addiction (r = 0.17, p = 0.212), smoking history (r = 0.07, p = 0.602), anxiety (r = 0.07, p = 0.602), depression (ρ = 0.07, p = 0.602), and quality of life (ρ = −0.05, p = 0.714).

## DISCUSSION

For this study, we translated the TSQ to Brazilian Portuguese, cross-culturally adapted it for use in Brazil, and evaluated its reliability. We found that all of the participants were satisfied with telemedicine and that the TSQ was reliable, rapidly applied, easily completed, and easy to understand.

The translation and cross-cultural adaptation of a questionnaire should follow the guidelines established by Beaton et al.^11^ and Wild et al.^12^ Depending on the complexity of a questionnaire, more than one translator can perform the translation and back-translation.^12^ When translating a questionnaire into the local language, it is recommended that the senior author of the original questionnaire be consulted to discuss and resolve questions regarding the interpretation of terms or phrases. In the cross-cultural adaptation phase, the version translated into the local language must be presented and discussed with persons with the same status the questionnaire was created, in order to determine which words best express what the questionnaire will assess. For this phase, a multidisciplinary team joined the investigators to discuss with patients which terms would be the best for the Brazilian Portuguese-language version of the TSQ. The translated version should then be back-translated by a different person, compared with the original version to evaluate similarity,^11^ ^,^ ^12^ and presented to the senior author of the original version for acceptance or suggestions.^11^ ^,^ ^12^ The translation and cross-cultural adaption of the TSQ for use in Brazil encompassed all of those phases. However, we were unable to contact the first or the senior authors of the original TSQ, despite sending messages via internet and attempting to contact the university. Nevertheless, we do not believe that the lack of contact limited the translation, given that the statements in the questionnaire were quite clear.

Few questionnaires assessing satisfaction with telemedicine can be found in the Brazilian literature. Studies similar to ours have used different questionnaires without clear descriptions regarding their development or translation. Although Dias et al.^17^ assessed satisfaction with telemedicine by questioning sociodemographic parameters, transportation, and travel time to the hospital, no description was presented regarding the number of patients satisfied with the care given by the health care professional. Those authors also did not describe the translation and cross-cultural adaptation. Although the TSQ has been previously used in Brazil for assessing the perspectives of legal guardians regarding consultations with patients under 18 years of age, only 7 of the 14 questions were asked and no information was given concerning the translation.^16^ Likewise, Brandão et al.^15^ applied a satisfaction survey for the use of immunobiologicals, although the questionnaire used was not specified.

The present study assessed reliability by applying the Brazilian Portuguese-language version of the TSQ at three different time points. Reliability is the repeatability or reproducibility of a measure or variable, and it is commonly assessed by applying a questionnaire at least twice.^25^ ^-^ ^27^ However, reliability increases with sample size and the number of questionnaire applications. For a sample size of 50 participants with three repetitions, the confidence intervals are narrower than, for instance, a sample of 15 participants with four repetitions. Therefore, the number of questionnaire applications is important in a study design to reduce the typical error.^22^ ^).^

Participants completed the TSQ by choosing one of the five answers on the Likert scale for each question. However, most of them stated that the answers “strongly disagree” and “disagree” and the options “strongly agree” and “agree” were similar and difficult to understand as different options. Considering that the answers “strongly agree” and “agree” expressed the same feeling (i.e., acceptance of what was considered in the question), we merged the two answers into one and found that most (89.9%) of the participants were satisfied with their telemedicine experience. Although Likert scales typically present five answer options, a different number of options can be considered. For example, in one study that cross-culturally adapted an instrument to investigate the perception of health professionals about teleconsultation, seven options were given.^28^ Likewise, five options were presented to patients undergoing treatment for type 2 diabetes,^4^ whereas three options were given to evaluate the responsiveness of a system by applying a usability script to three devices^29^: notebooks, tablets, and smartphones. It is possible that the level of education influenced the number of answer options; that is, participants with a high level of education might be more capable of sorting out a more precise answer than are those with no formal education. Nevertheless, we observed no differences among different education levels in terms of the answers chosen.

For questions 12, 13, and 14 of the TSQ, which refer directly to satisfaction with telemedicine, the positive response rate was high (87.7%, 94.3%, and 94.3%, respectively) after three interviews. The reasons why patients were satisfied with telemedicine were assessed through questions 7, 9, 10, and 11, which also presented high reliability (90.1%, 94.3%, 84.9%, and 90.6%, respectively). The ICC analysis was hampered by the large percentage of answers grouped into two responses, considering the five Likert scale options.

The novel finding of this study was the high rate of satisfaction with telemedicine by our patients. Telemedicine has been used for over 30 years,^30^ and the COVID-19 pandemic recently led to in its adoption worldwide. With the closure of the outpatient clinic, virtual consultations became an alternative for communication between health care professionals and patients. Our smoking cessation clinic maintained the group orientation through telemedicine, but information on patient satisfaction or whether they would prefer to go back to the previous system (in-person group meetings) was lacking. Despite the high satisfaction rate, our results may apply only to certain consultations. Knowledge about the physical health of patients was optional in the smoking cessation group, because the meetings focused on advice and reinforcement techniques.

One limitation of our study was the initial refusal of participants to respond to calls and questions at three different time points to answer the same questions; they considered the repetition unnecessary. This was mitigated by explaining the reasons for the repetition and emphasizing the need for appropriate guidance to increase the reliability of the study. Another limitation was the application of the TSQ to patients who required only guidance and motivation to stop smoking. Therefore, other specialties should use the Portuguese-language version of the TSQ that has been cross-culturally adapted for use in Brazil to determine whether patients were satisfied with telemedicine. A third possible limitation was the lack of a test-retest reliability analysis.

Despite its development in 2002 and use in various studies, the TSQ is not widely used in Brazil, probably because previous translations were not performed in accordance with the standards.^11^ In addition, the use of telemedicine became widespread in Brazil in 2020 (during the COVID-19 pandemic) and was regulated only in 2022.^31^ With the increasing development of telemedicine in Brazil, the TSQ is a reliable tool to assess the level of satisfaction of patients regarding health care services.

One of the strengths of the present study was the large sample size and application of the TSQ at three different time points. This design increased the reliability of results. Another strength was the high reliability regarding satisfaction with telemedicine. In addition, no significant difference was observed between the TSQ score and the variables assessed, indicating that it may be applied to any individual, regardless of age, gender, education level, socioeconomic class, symptoms of depression or anxiety, and quality of life.

The Portuguese-language version of the TSQ, cross-culturally adapted for use in Brazil, appears to be easily and quickly applied, and patients considered the use of telemedicine acceptable in the smoking cessation group meetings. This acceptability might have been influenced by the fact that telemedicine is reliable, saves time, provides adequate attention, and meets health care needs. Applying the TSQ at three different time points, with three answer options, showed high test-retest reliability.
